# INTERPROFESSIONAL SOCIALIZATION AND VALUING SCALE FOR HEALTH PROFESSIONAL STUDENTS WORKING WITH OLDER ADULTS

**DOI:** 10.1093/geroni/igz038.1081

**Published:** 2019-11-08

**Authors:** David Cicala, Nicholas Ang, Jennifer Mendez, Shanique Brown, Martha Schiller

**Affiliations:** 1 Wayne State University, Detroit, Michigan, United States; 2 Wayne State University School of Medicine, Detroit, Michigan, United States; 3 Wayne State University, Detroit, United States

## Abstract

Interprofessional education allows students from two or more professions the opportunity to collaboratively practice patient centered care. Given the importance of interprofessional education in helping individuals become effective team members and understanding their value to a healthcare team, there is a need to evaluate the experiences. The Interprofessional Socialization and Valuing Scale (ISVS), aims to measure self-perceived experiences with interprofessional collaborative teamwork, including the ability, value, and comfort in working with others. The Wayne State University Interprofessional Team Visit (IPTV) program is an older adult home visit program that places pharmacy, social work, occupational therapy, nursing, and medical students in teams. Students form teams of 3 different disciplines and interview the older adult to assess various aspects of health and wellbeing. In order to evaluate the interprofessional educational experiences of the students, they are given a pre- and post- survey utilizing the ISVS tool. 18 questions pertaining to perceptions of what students have learned about working with professionals from other disciplines. Students respond to each statement using a 7-point scale with 1 = “Not at All” and 7 = “To a Very Great Extent.” Statistical analysis is conducted in order to compare pre- to post-surveys and also assess differences between groups. It is found that ISVS scores increase from pre- to post-survey, second year medical students and third year pharmacy students feel more comfortable working in teams, and teams consisting of these two have higher average scores.

